# Genotyping of Infectious Laryngotracheitis Virus (ILTV) Isolates from Western Canadian Provinces of Alberta and British Columbia Based on Partial Open Reading Frame (ORF) a and b

**DOI:** 10.3390/ani10091634

**Published:** 2020-09-11

**Authors:** Catalina Barboza-Solis, Ana Perez Contreras, Victor A. Palomino-Tapia, Tomy Joseph, Robin King, Madhu Ravi, Delores Peters, Kevin Fonseca, Carl A. Gagnon, Frank van der Meer, Mohamed Faizal Abdul-Careem

**Affiliations:** 1 Faculty of Veterinary Medicine, University of Calgary, Health Research Innovation Center 2C53, 3330 Hospital Drive NW, Calgary, AB T2N 4N1, Canada; catalina.barboza@ucalgary.ca (C.B.-S.); ana.perezcontreras@ucalgary.ca (A.P.C.); victor.palominotapia@ucalgary.ca (V.A.P.-T.); fjvander@ucalgary.ca (F.v.d.M.); 2 Animal Health Centre, Ministry of Agriculture, Abbotsford, BC V3G 2M3, Canada; tomy.joseph@gov.bc.ca; 3 Agri Food Laboratories, Alberta Agriculture and Forestry, Edmonton, AB T6H 4P2, Canada; blking@telus.net; 4 Animal Health and Assurance, Alberta Agriculture and Forestry, Edmonton, AB T6H 4P2, Canada; madhu.ravi@gov.ab.ca (M.R.); delores.peters@gov.ab.ca (D.P.); 5 Provincial Laboratory for Public Health, Calgary, AB T2N 4W4, Canada; kevin.fonseca@albertaprecisionlabs.ca; 6 Swine and Poultry Infectious Diseases Research Center (CRIPA), Faculté de médecine vétérinaire, Université de Montréal, 3200 Sicotte, Saint-Hyacinthe, QC J2S 2M2, Canada; carl.a.gagnon@umontreal.ca

**Keywords:** infectious laryngotracheitis, genotyping, Canada, Sanger sequencing, live attenuated vaccine

## Abstract

**Simple Summary:**

Infectious laryngotracheitis virus is an economically important acute upper respiratory tract disease in chickens. To control the disease, there are two types of vaccines commercially available, the recombinant viral vector and the live attenuated vaccines. The live attenuated vaccines are effective in disease control, but because of their residual virulence, they can replicate, cause disease, and revert to their original virulent form. Strains of the virus can be categorized as vaccine-related or wild type related. Information is scarce on the type of strains that are circulating in Canada. This study aims to discriminate between wild type and vaccine strains associated with infectious laryngotracheitis cases in the provinces of Alberta and British Columbia between the years 2009–2018. To accomplish this objective, the sequencing of two specific partial genes was performed. As a result, 27 samples from Alberta, and 5 samples from British Columbia were successfully sequenced. From the total samples, ~85% were related to vaccine strains and the rest categorized as wild type. These results reinforce the concern on current practices surrounding vaccination and the need to implement better biosecurity measures.

**Abstract:**

Infectious laryngotracheitis virus (ILTV) causes an acute upper respiratory disease in chickens called infectious laryngotracheitis (ILT). Live attenuated vaccines are effective in disease control; however, they have residual virulence, which makes them able to replicate, cause disease and revert to the original virulent form. Information is scarce on the molecular nature of ILTV that is linked to ILT in Canada. This study aims to determine whether isolates originating from ILT cases in Western Canada are a wild type or vaccine origin. Samples submitted for the diagnosis of ILT between 2009–2018 were obtained from Alberta (AB, *n* = 46) and British Columbia (BC, *n* = 9). For genotyping, a Sanger sequencing of open reading frame (ORF) a and b was used. A total of 27 from AB, and 5 from BC samples yielded a fragment of 1751 base pairs (bp). Three of the BC samples classified as group IV (CEO vaccine strains) and 2 as group V (CEO revertant). Of the AB samples, 22 samples clustered with group V, 3 with group VI (wild type), and 2 with group VII, VIII, and IX (wild type). Overall, 17 non-synonymous single nucleotide polymorphisms (SNPs) were detected. Further studies are underway to ascertain the virulence and transmission potential of these isolates.

## 1. Introduction

The etiological agent responsible for infectious laryngotracheitis (ILT), an acute upper respiratory tract disease in chickens, is the *Gallid alphaherpesvirus 1* (infectious laryngotracheitis virus (ILTV)). ILT is most commonly seen in large, high-density production areas where it is horizontally transmitted [1]. Enzootic forms of ILT show high morbidity (90–100%), and depending on the infecting strain, more variable mortality (5–70%) can be observed [2]. Even though viremia has not yet been described, in vitro experiments indicated that ILTV can infect macrophages, potentially leading to infection of non-respiratory sites [3]. The incubation period following natural infection ranges from 6 to 12 days [4,5], while experimental intratracheal challenge usually results clinical signs within 2 to 4 days [6]. Lifelong latent infections will be established following the acute phase. This was first described in the trachea [7] and further demonstrated in the recent year [8]. However, the predominant site of latency is the trigeminal ganglia (TG) [9,10,11,12].

Pathogenicity may differ between isolates, but typical acute clinical signs are conjunctivitis, nasal discharge, drop in egg production, sinusitis resulting in decreased body weight gain, and predisposition to other respiratory pathogens [13]. In the case of severe infection, inflammation, necrosis, hemorrhage, and ulceration of upper respiratory tract are found on pathology; furthermore, the formation of diphtheritic membranes may obstruct the airways resulting in death from asphyxia [14]. ILT can affect chickens at any age, although most often at four weeks of age, or even younger [15].

ILT is endemic in backyard flocks of Canada [16], and infrequent ILT outbreaks are recorded in commercial poultry operations in many parts of the country [13,17,18,19], most recently, (2017–2019) an ILT outbreak in the provinces of Ontario and Québec involved commercial chickens [20,21].

There are two types of ILTV vaccines commercially available: A live attenuated and a recombinant viral vector. Live attenuated virus vaccines can be either embryo (chicken embryo origin, CEO) or tissue culture passaged (tissue culture origin, TCO) and are satisfactory choices in ILT control [22]. On the downside, their ability to replicate and residual virulence can still cause disease [23,24]. Outbreaks of ILT in the United States (US) were traced back to CEO vaccines [24]. In Canada, the TCO and CEO vaccines are licensed and manufactured and distributed by Merck Animal Health (Madison, NJ, USA) and Merial Select Inc. (Gainesville, GA, USA) respectively. On the other hand, recombinant viral vector vaccines are safer. Although they are more expensive and when compared to live attenuated vaccines, studies have shown that they are not as effective as the CEO vaccine in reducing viral shedding [25,26,27]. Fowl poxvirus (FPV) and herpesvirus of turkeys (HVT) are the two viral vectors utilized in these recombinant vaccines. The FPV has the glycoprotein B and unique long (UL) 32 gene from ILTV (licensed in Canada and manufacture by CEVA Biomune, Lenexa, KS, USA) [28]. The HVT carries the glycoprotein I and D (licensed in Canada and manufactured by Merck Animal Health, Madison, NJ, USA), and a more recent one has the glycoprotein B (licensed in Canada and manufactured by CEVA Biomune, Lenexa, KS, USA) [29]. Although all these vaccines are licensed in Canada, usage varies in the different provinces. For example, in Alberta (AB), recombinant viral vector vaccines and TCO vaccines are recommended by the Ministry of Alberta Agriculture and Forestry since 2009.

There is an array of molecular assays available for the purpose of differentiating wild-type strains from vaccine viruses. One of the most common assays used is the polymerase chain reaction (PCR)-restriction fragment length polymorphism (RFLP) that targets multiple ILTV genome regions [30,31,32,33]. However, this assay is time-consuming and expensive. Recently partial sequencing (using Sanger sequencing technology), of the gene open reading frame (ORF) a and b [34], which are unique to the Iltovirus genus [35], was developed as a faster alternative. This method enables the differentiation of vaccine and wild type ILTV strains. In this way, strains are classified as either TCO vaccine-related, CEO vaccine, CEO revertant, or wild type through six single nucleotide polymorphisms (SNPs). The ability to differentiate circulating wild type and modified live vaccine viruses, is essential for ILT control [36].

Genomic surveillance of the circulating ILTV strains in a geographical area is essential for the development of control measures [37]. The most recent genetic data of ILTV strains in Canada dates back to 2006 [13], and this previous study examined samples from a 2004–2005 outbreak of ILT in the Niagara Peninsula in Southern Ontario. This later study demonstrated that both wild type and CEO vaccine-derived ILTV strains were circulating.

The objective of this study was to characterize ILTV isolates associated with several ILT cases in Western Canada recorded during 2009–2018, to discriminate between wild type and vaccine strains.

## 2. Materials and Methods

### 2.1. ILTV Isolates

Between 2009 and 2018, qPCR positive clinical ILT samples were collected along with their background information from Agri Food Laboratories, Alberta Agriculture and Forestry, AB (*n* = 46) (Table S1) and from Animal Health Center, British Columbia (BC, *n* = 9) (Table S2). The samples obtained from AB were comprised of tracheas and tracheal swabs in viral transport medium. Samples obtained from BC had been propagated in chicken embryo kidney cells (CEKC) from tracheal swabs. Tracheal swabs samples and cell culture samples were aliquoted and stored at −80 °C along with the tissues until further processing.

### 2.2. ILTV Propagation

The samples that were negative in the PCR assay (*n* = 7) for sequencing, were propagated either in chicken embryo liver cells (CELIC) or by inoculation on the chorioallantoic membranes (CAMs) (Table S3). The SPF chicken eggs were obtained from the Canadian Food Inspection Agency (CFIA), Ottawa, ON, Canada.

The use of embryonated eggs for ILTV propagation was approved by the Health Science Animal Care Committee (HSACC) of the University of Calgary, Alberta, Canada (Protocol number: AC19-0013).

### 2.3. ILTV Propagation in Chicken Embryo Liver Cell

The CELIC were prepared using liver tissues harvested from 14-day-old chicken embryos [38]. The maintenance media comprised of Dulbecco’s modified Eagle medium (DMEM) containing 10% fetal bovine serum (FBS, Gibco, Carlsbad, CA, USA), and 100 U/mL penicillin and 100 μg/mL streptomycin (Gibco, Carlsbad, CA, USA). The cells were incubated at 37 °C with 5% CO2, and at >80% confluence, they were infected with 100 μL of the ILTV isolates using DMEM containing 2% calf serum (CS) and 100 U/mL penicillin and 100 μg/mL streptomycin. The inoculum consisted of trachea homogenized in 1 mL of phosphate-buffered saline (PBS, Lonza, Walkersville, MD, USA) and 15 μL of antibiotic (100 U/mL penicillin and 100 μg/mL streptomycin). At five days following infection or when the cytopathic effect was extensive, the cells were harvested by freezing and thawing for 30 min three times. The propagated samples were aliquoted and kept at −80 °C.

PCR that targets the ORF a and b partial sequence (USDA reference genome coordinates 21,703–23,895) was used to verify successful virus propagation. Samples that remained negative by PCR after three cell passages, were subsequently propagated in embryonated eggs from the original sample.

### 2.4. CAM Inoculation

For the propagation of ILTV in embryonated chicken eggs, 10-day-old SPF eggs were used. Briefly, a small hole was drilled in the air cell, and the egg was placed horizontally, then the air was drawn out with a rubber bulb to create a new, artificial, air cell in which the inoculum was placed using a 25-gauge needle [38]. After five days of inoculation, the eggs were placed in a refrigirator for 24 h. The infected CAMs were extracted and thoroughly minced, then homogenized with a mini homogenizer, and aliquots were stored at −80 °C. After the CAMs extraction, they were observed for the presence of pock lesions, however none were observed.

### 2.5. DNA Extraction

DNA extraction from tracheal tissues (*n* = 10), tracheal swabs (*n* = 15), and cell culture supernatants (*n* = 7) were performed using QIAamp^®^ DNA Mini Kit (Qiagen, Hilden, Germany) based on manufacturer’s instructions. Briefly, tracheas were homogenized with a mini homogenizer and aliquoted. The volume utilized for the DNA extraction was 200 μL. Later, the extracted DNA was quantified with the Nanodrop 1000 spectrophotometer (ThermoScientific, Wilmington DE, USA) with absorbance at 260 nm.

### 2.6. PCR and Amplicon Purification

Two separate PCR reactions were performed targeting two different regions of ORF a and b. The first reaction, made with a total volume of 50 μL, was based on already published protocol with modifications [34]. The reaction included 200 nM of primers ILTVF1_F2 (5′ TTTTGTGCTCATCGCTGTTC3′) and ILTV 1R_R1 (5′CAGCGTTGTGAATT GCTTGT3′) (USDA reference genome coordinates 21,703–23,895) in a reaction containing 2.5 U Taq DNA polymerase (non-high-fidelity enzyme) per reaction, 0.2 mM of 10 mM dNTP mix, 1X of 10X PCR Buffer—Mg, 1.5 mM of 50 mM MgCl2 and 100 ng of DNA template (Invitrogen, Burlington, ON, Canada) and resulted in a 1751 bp amplicon (Figure S1). The thermocycler conditions were: Initial denaturation at 94 °C for 2 min, followed by 40 cycles of a three-step amplification protocol: Denaturation at 94 °C for 30 s, annealing at 58 °C and elongation at 68 °C for 60 s each.

**Figure 1:**
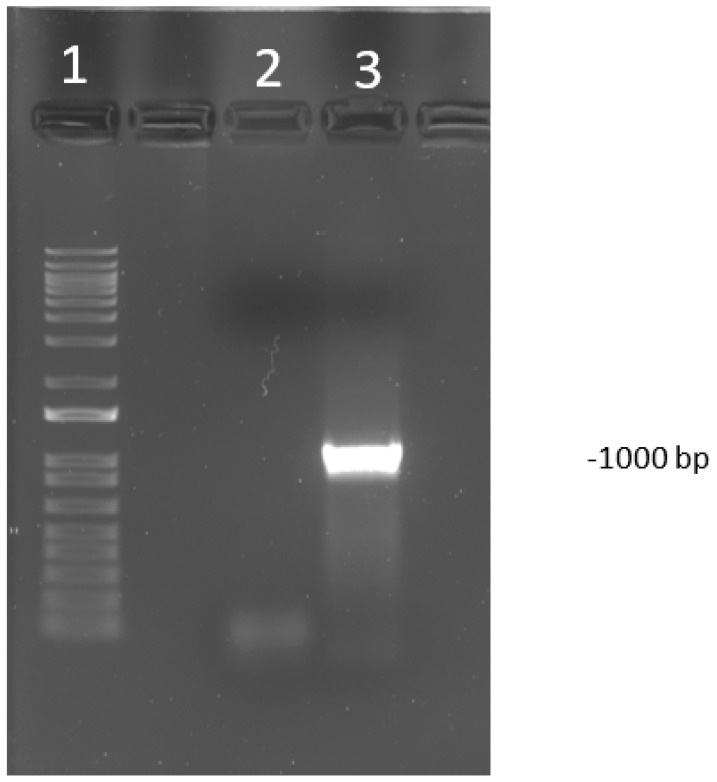
Visualization of polymerase chain reaction (PCR) product run on a 1% agarose gel of PCR targeting open reading frame (ORF) a and b (USDA reference genome coordinates 22,364–23,364). The amplicon size is 1000 bp. The DNA ladder used was 1 kilo base pairs plus (kb+). Lane 1 is the DNA ladder. The lane labeled 2 is a negative control. Lane labeled as 3 is a known positive sample to an infectious laryngotracheitis virus (ILTV).

However, after sending the samples for sequencing, the laboratory reported that some samples presented secondary structures or loss of signal, making the sequencing incomplete. Thus, a second reaction targeting a 1000 bp amplicon within the first target was performed to fill the gaps in the first obtained sequence (Figure 1). This reaction was similar but with a different set of primers; ILTVF (5′ CGAATGCATCCTTAGACGGG 3′) and ILTVR (5′ AGCTCGAGAAATTGCAGCG 3′) (USDA reference genome coordinates 22,364-23,364). The primers were designed using the Primer3 web version 4.0.0 with the default settings (https://primer3plus.com/primer3web/primer3web_input.htm). For this second reaction, thermocycler conditions were: Initial denaturation at 94 °C for 2 min, followed by 40 cycles of a three-step amplification protocol: Denaturation at 94 °C for 30 s, annealing at 60 °C and elongation at 68 °C for 60 s each. All PCR products were purified using QIAquick^®^ PCR Purification Kit (Qiagen, Hilden, Germany).

After purification, samples were sent for Sanger sequencing at the University of Calgary Core DNA Services (Calgary, AB, Canada). This facility used an Applied Biosystems 3730xl (96 capillary) genetic analyzer.

### 2.7. Phylogenetic Analysis

**Figure 2:**
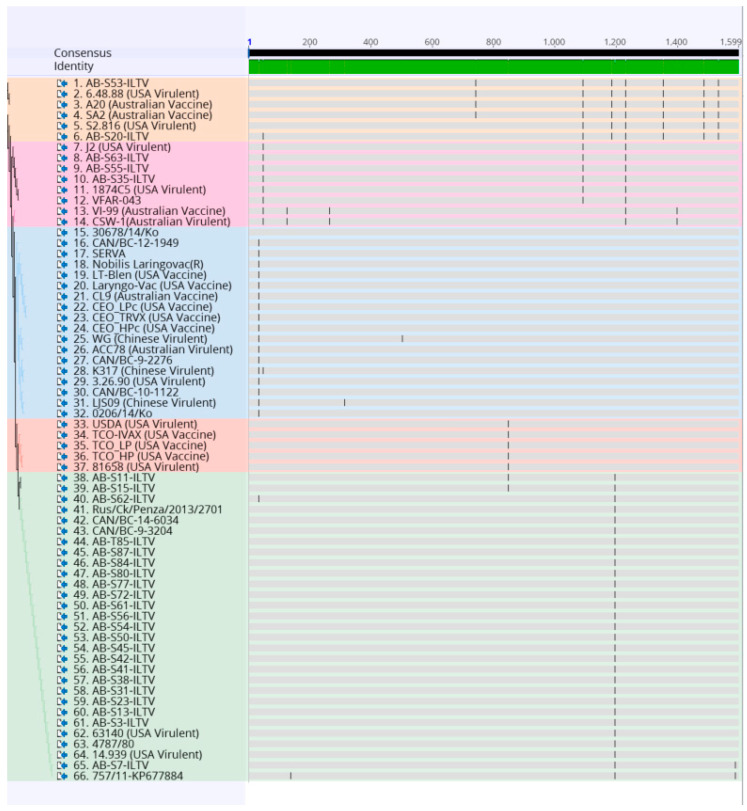
Alignment of the nucleotide sequences of 32 Canadian ILTV strains and 34 ILTV reference strains using MUltiple Sequence Comparison by Log-Expectation (MUSCLE) and Geneious software package. Vertical lines indicate single nucleotide polymorphism (SNP) positions. Samples are color-coded as the phylogenetic tree.

Sequences were subjected to the Basic Local Alignment Search tool (BLAST) analysis in NCBI (https://blast.ncbi.nlm.nih.gov/Blast.cgi) to confirm nucleotide identity. A sequence was generated by analyzing the forward and reverse sequences of the first reaction obtained with each sample using the Geneious version 10.0.9 (Biomatters Ltd., Auckland, New Zealand). The sequences obtained from the second PCR reaction were used when a big gap between the reverse and forward sequence of the first reaction was found. The nucleotide sequence of the 32 partial ORF a and b gene segments obtained through this study were aligned with 34 reference ILTV strains downloaded from the GenBank (https://www.ncbi.nlm.nih.gov/genbank/) (Table S4). The alignment was performed using MUltiple Sequence Comparison by Log-Expectation (MUSCLE) on Geneious version 10.0.9 (Biomatters Ltd., Auckland, New Zealand) (Figure 2). To do the analysis, some sequences had to be reduced in size (1599 bp), so that a larger number of samples could be added to the analysis. The reduction was made by ensuring that the critically informative single nucleotide polymorphism (SNPs) necessary for genotyping, mention in the previous study [34], would be included.

A phylogenetic tree was generated with Phylogenetic inferences using Maximum Likelihood (PHYML) [39] in Geneious set to 1000 bootstrap replicates using the concatenated sequences.

**Figure 3:**
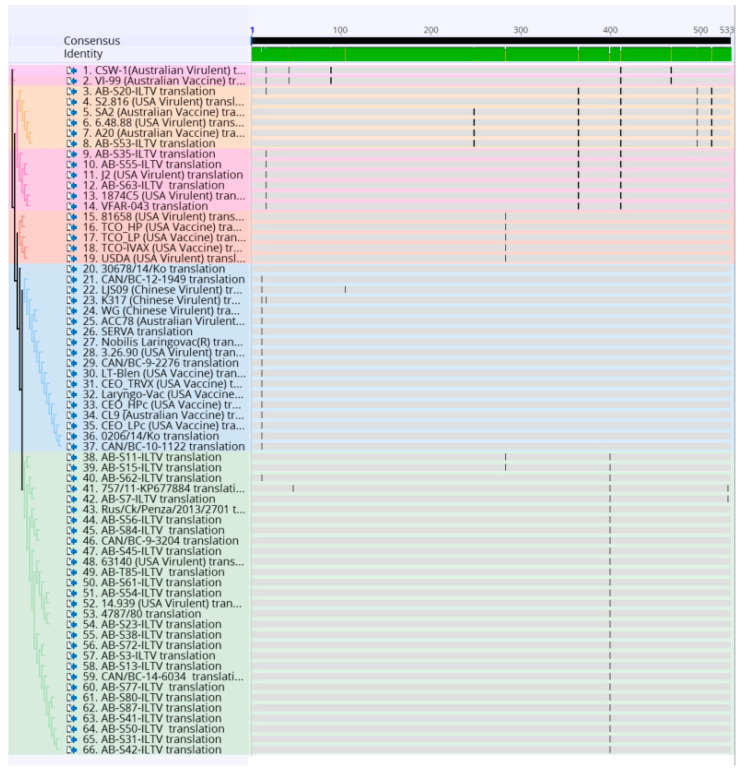
Alignment of the amino acid sequence of 32 Canadian ILTV strains and 36 ILTV reference strains using MUSCLE and Geneious software package. Vertical lines indicate nucleotide change positions. Samples are color-coded as the phylogenetic tree.

To identify single nucleotide polymorphisms that led to non-synonymous substitutions, all ILTV strains were translated into amino acids and realigned using multiple sequence alignment MUSCLE on the Geneious version 10.0.9 (Biomatters Ltd., Auckland, New Zealand) (Figure 3).

The nucleotide sequences were uploaded to the GenBank (accession numbers are in Table 1).

## 3. Results

The background information relevant to the analyzed ILT samples is depicted in Table 1. The BC samples originated from commercial broiler and layer flocks, the only sample CAN/BC-10-1122, was accompanied with a vaccination history. All the AB samples originated from chickens from non-commercial farms (in AB, less than 2000 broilers and 300 layers per household/per year are allowed to raise without quota, and defined as non-commercial poultry flocks), only sample AB-S15-ILTV came from a vaccinated flock. Samples obtained from AB came from birds with different ages (1.5 to 24 months), as well as samples obtained from BC (36 days to 12 weeks).

Conventional PCR assays conducted targeting two areas of ORF a and b, and the resulting products that were sequenced are shown in Figure 1 and Supplementary Figure S1.

From the initial 55 samples, only 32 were successfully sequenced. The other 23 were either negative in the PCR for sequencing, or the results after the sequencing were of poor quality. Of the 32 PCR positive samples, 27 were from AB, and 5 were from BC (Table 1).

The resulted nucleotide sequences of the 32 ORF a and b gene segments were aligned with 34 reference ILTV strains and illustrated in Figure 2. Further analysis of amino acid sequences of current ORF a and b segments and reference amino acid sequences are shown in Figure 3. Overall, 17 single nucleotide polymorphisms (SNPs) that lead to non-synonymous substitutions were found in the processed samples and references (Table 2).

**Figure 4:**
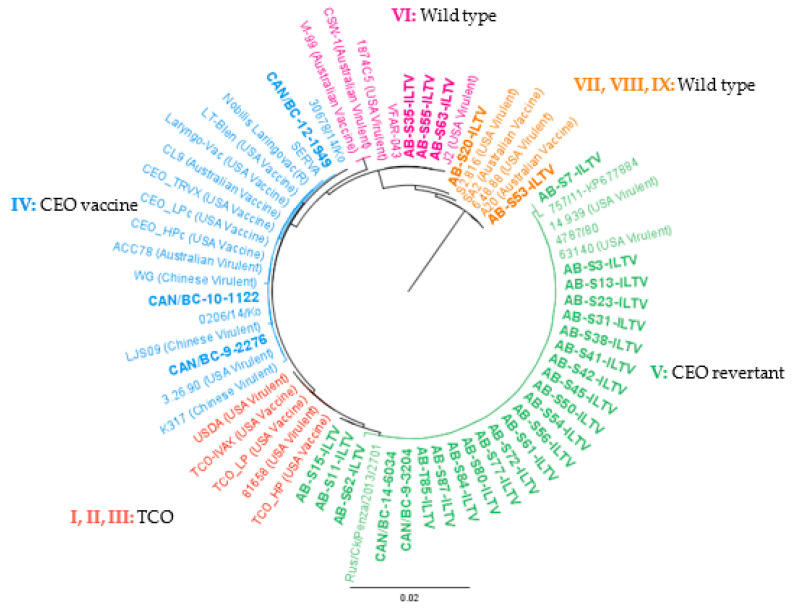
PHYML phylogenetic tree with nucleotide sequences of 66 partial ORF a and b of ILTV. The cladogram includes 34 ILTV reference strains downloaded from the GenBank and 32 ILTV Canadian strains characterized in the current study in bold (AB = 27, BC = 5). The genotype nomenclature used is based on [34]. Geneious version 10.0 was created by Biomatters. Available from http://www.geneious.com.

These ILTV strains could be grouped into four previously published clusters [34], as shown in Figure 4. Of the BC ILTV strains, three were classified in group IV (CEO vaccine), and the remaining two in group V (CEO revertant). Of the AB isolates, 22 clustered in group V, 3 in cluster VI (wild type), and 2 in cluster VII, VIII, and IX (wild type) (Figure 4).

Of the totality of the processed samples, none clustered in group I, II, and III (TCO related). Of the samples from AB, none clustered in group IV, whereas three of the BC samples did. The nucleotide identity of ILTV isolates classified in group IV varied from 99.9% to 100%. The BC samples showed the lowest nucleotide identity (99.9%) to the rest of the strains in the group.

The ILTV clustering in group V were 99.8–100% similar. The Italian strain 757/11 was the most distant of the ILTV in group V; however, AB-S7-ILTV showed a 99.9% identity to the Italian strain 757/11. Samples AB-S11-ILTV and AB-S15-ILTV were genetically identical and were 99.9% similar to the rest of the strains in group V. It should be noted that both samples were from the same year and could be the same ILTV strain, however, analysis of more variable genome regions are necessary for confirmation. In group VI, all the sequences obtained were identical except for the Australian strains (99.8%). In the final clusters (VII, VIII, and IX), AB-S53-ILTV was identical to Australian vaccine strains and US virulent strain 6.48.88. In the case of sample AB-S20-ILTV, it was more similar to the US virulent strain S2.816 (99.9%).

## 4. Discussion

The aim of the current study was to characterize the ILTV isolates that originated from various ILT outbreaks in chickens raised in Western Canada. The molecular epidemiology of recent outbreaks of ILTV in chickens from Western Canada is poorly defined. The current study genetically characterized parts of the ORF a and b genes of 32 ILTV isolates collected during ILT outbreaks in Western Canada. It evaluated mostly non-vaccinated flocks, on which the predominant reason for outbreaks were strains related to CEO vaccine strains. These results contribute to the ongoing discussion related to the safety of using the CEO vaccine and the use of safer options like the recombinant viral vector vaccines. Additionally, these findings support the need to implement better biosecurity measures and effective vaccination strategies in backyard flocks.

ILT outbreaks, related to TCO vaccine strains, are globally reported, but not as frequent as CEO related ones [24,40,41,42]. In our study, none of the examined AB and BC ILTV isolates were identify as TCO vaccine strains. This is an interesting finding since TCO vaccines are recommended by the Ministry of Alberta Agriculture and Forestry and used by flock owners since 2009. Our findings are in agreement with previous studies that recorded lower transmissibility of TCO vaccine strains compared to CEO vaccines [30,40,43]. It has also been shown that TCO vaccines do not revert to virulence like CEO vaccines following the passage for 20 times in vivo [44]. However, this could be due to low pathogenicity or low transmission of TCO vaccine strains into non-commercial poultry, there is evidence that shows that TCO vaccine strains acted as parental strains contributing to the emergence of recombinant strains with higher virulence [41]. To be able to determine if the currently studied ILTV strains are the product of recombination, full genome sequence or sequencing of a bigger genomic region is needed.

On the other hand, CEO related strains are identified in recent outbreaks of ILT in the US [25], and in Ontario, Canada [13]. In the current study, CEO vaccine ILTV strains were identified in samples from BC but not from AB, possibly because the Ministry of Alberta Agriculture and Forestry recommends the use of TCO and not CEO for backyard poultry producers in the last decade. In addition, Egypt, Korea, and Australia have also reported that some ILTV strains are the result of recombination, with at least one of the parental strains being a CEO vaccine virus [41,45,46,47]. To ascertain whether ILTV strains circulating in BC are a product of recombination events, full genome sequence analysis is necessary. Although CEO vaccine viruses are attenuated, bird-to-bird passages can enable the reversion to a virulent state, which can cause disease [44]. Most of the AB samples classified as CEO revertant (Group V) even though CEO vaccines were discouraged from being used for decades. Similarly, a recent study in Argentina found circulating strains were CEO revertants, even though the use of CEO vaccines has been prohibited for the last 10 years [42]. The hypothesis was that the CEO vaccine strains may have still been circulating, due to various bird-to-bird passage gaining virulence back [44], leading to ILT outbreaks [42]. In AB, ILTV is endemic in non-commercial flocks, and it is possible that CEO vaccines used a decade back are still circulating because ILTV infection is a lifelong infection (with an established carrier state), and multi-age birds are common in backyard flocks, providing a perfect scenario for the ongoing spread of the CEO related ILTV. These poultry flocks could potentially be constant sources of this virus for naïve chickens, since the movement of birds through long distances, particularly the rare breeds for various shows and competitions, is common. An alternative explanation may be the transmission of CEO vaccines or revertant strains from neighboring provinces with heavy use of CEO vaccines, which was demonstrated in Brazil [9]. Studies have shown that the combined usage of live attenuated and recombinant vaccines may produce long term protection against CEO revertant strain 63140 [48]. However, to the best of the knowledge of the authors, short term protection of combination or single vaccines against CEO revertant strains have not been studied.

In AB, five of the ILTV isolates were wild type and not related to ILT live attenuated vaccines. It is well established that live attenuated ILT vaccines prevent clinical signs, but do not completely protect birds from wild type ILTV replication [9,25]. The ILT outbreaks caused by wild type ILTV strains are reported in different countries, although to a lesser degree than CEO vaccine-related ILT outbreaks [13,24,49]. The findings of this study are in agreement with the previous study done in Ontario [13], wild type ILTV caused outbreaks are not uncommon in Canada, but in a lesser degree than vaccine-related ILT. It would be interesting to compare our data to data of more recent outbreaks in commercial flocks in Québec.

Through our analysis, we were able to detect 17 SNPs that were non-synonymous. Previously published data reported six SNPs in positions 50, 848, 1196, 1231, 1533, and 1534 [34] that were critically informative to phylogenetically separate the ILTV groups are also identified in our samples. Additionally, 11 different SNPs were found, from which four were shared by specific groups. One was shared by all viruses of group IV in position 34. Two that were found only in group VII, VIII, and IX, and one that was shared by all wild type viruses in position 742. The other seven SNPs were unique to different strains not shared within their group (Table 2). At comparing CEO vaccine strains and CEO revertant, they differ in two different mutations. CEO vaccine has a transversion mutation in position 34 (Lys12Gln) that is unique to the genotype; on the other hand, CEO revertant has a transversion mutation in position 1196 (Gln399Pro) that is unique to the cluster. Wild type clusters share two common SNPs in position 1091 (Val364Ala) and 1231 (Gly411Cys). However, cluster VI has a unique SNP in position 50 (Ala12Val), and cluster VII, VIII, IX has three unique SNPs in position 742 (Leu105Ser), 1487 (Lys496Arg), and 1533 (Ala512Thr).

A previous study has found that mutations on 12 different genes (ORF c, ORF e, ORF f, UL23, UL39, UL36, UL26, UL28, UL17, UL27, UL10, and UL8) that are exclusive to a CEO revertant strain (63140) in comparison to CEO vaccines from the US; however, ORF a and b were not included in this study [1]. Additionally, it describes seven non-synonymous substitutions in five genes (ICP4, UL54, UL5, UL38, and UL8) involved in virulence/attenuation phenotypes for the CEO group and three mutations in the UL5 gene for the TCO group.

The ORF a and b are two of the five unique genes in the UL region that characterize the Iltovirus genus from other alpha herpesviruses [35]. These specific genes encode a protein product of 40 kilodaltons (kDa) in the case of ORF a and 34 kDa in the case of ORF b [50]. According to database searches, they seem to have no significant homologies to other viral proteins and appear to not have conserved motifs that may point to function or location within virions [51]. Nonetheless, RNA analysis indicates that they might express during virus infection. It was later found that these genes are dispensable for ILTV replication in tissue culture, but might aid in immune evasion or species specificity [52]. Another study suggested that ORF a and b function for virus replication might be important but redundant [35]. It then would be difficult to ascertain what these specific amino acid changes might do in terms of virulence or immune evasion, as it is still unclear the specific role these genes have. However, if or when, further studies are done to ascertain the functions of these specific genes, these mutations might provide useful information to determine differences between genotypes.

## 5. Conclusions

In conclusion, the majority (>80%) of the ILT outbreaks in backyard poultry flocks from AB were found to be related to CEO revertants (22 of 27). Although CEO vaccines have not been used for decades in AB, it is possible that underground transmission of CEO revertants in unvaccinated chickens had been taking place for decades resulting in these recent ILT outbreaks. On the other hand, we also determined that wild-type outbreaks are not uncommon and can be observed in unvaccinated flocks. Outbreaks in backyard flocks could be a source of transmission to commercial operations; however, additional studies and information is necessary to trace transmission between commercial and non-commercial operations. Moreover, further analysis of the CEO vaccine viruses that are isolated from BC flocks is necessary to determine if these virus strains are the product of recombinant events. More studies are underway to ascertain the virulence and transmission potential of these Canadian ILTV isolates. It is also important to generate molecular epidemiology data relevant to more recent outbreaks of ILT in commercial chickens in Québec.

## Figures and Tables

**Table 1:** Classification of ILTV isolates from AB and BC is arranged by the year. The table includes the type of flock, province of origin, and the vaccination history. Samples name and genotype have been color-coded as the phylogenetic tree.

Isolates	Origin	Year	ORF a and b Gene Genotyping	Province	GenBank Accession Number	Vaccinated
CAN/BC-9-2276	Broiler	2009	IV	British Columbia	MT639625	No
CAN/BC-9-3204	Broiler	2009	V	British Columbia	MT639621	Unknown
CAN/BC-10-1122	Layer	2010	IV	British Columbia	MT639626	Yes
CAN/BC-12-1949	Broiler	2012	IV	British Columbia	MT639627	No
AB-S3-ILTV	Backyard chicken	2013	V	Alberta	MT639620	No
CAN/BC-14-6034	Layer	2014	V	British Columbia	MT639619	Unknown
AB-S7-ILTV	Backyard chicken	2014	V	Alberta	MT639603	No
AB-S11-ILTV	Backyard chicken	2014	V	Alberta	MT639601	No
AB-S13-ILTV	Backyard chicken	2014	V	Alberta	MT639605	No
AB-S15-ILTV	Backyard chicken	2014	V	Alberta	MT639602	Yes
AB-S20-ILTV	Backyard chicken	2015	VII, VIII, IX	Alberta	MT639631	No
AB-S23-ILTV	Backyard chicken	2015	V	Alberta	MT639606	No
AB-S31-ILTV	Backyard chicken	2015	V	Alberta	MT639607	No
AB-S35-ILTV	Backyard chicken	2015	VI	Alberta	MT639628	No
AB-S38-ILTV	Backyard chicken	2015	V	Alberta	MT639608	No
AB-S41-ILTV	Backyard chicken	2016	V	Alberta	MT639609	No
AB-S42-ILTV	Backyard chicken	2016	V	Alberta	MT639610	No
AB-S45-ILTV	Backyard chicken	2016	V	Alberta	MT639611	No
AB-S50-ILTV	Backyard chicken	2016	V	Alberta	MT639613	No
AB-S53-ILTV	Backyard chicken	2016	VII, VIII, IX	Alberta	MT639632	No
AB-S54-ILTV	Backyard chicken	2016	V	Alberta	MT639622	No
AB-S55-ILTV	Backyard chicken	2016	VI	Alberta	MT639629	No
AB-S56-ILTV	Backyard chicken	2017	V	Alberta	MT639604	No
AB-S61-ILTV	Backyard chicken	2017	V	Alberta	MT639623	No
AB-S62-ILTV	Backyard chicken	2017	V	Alberta	MT639624	No
AB-S63-ILTV	Backyard chicken	2017	VI	Alberta	MT639630	No
AB-S72-ILTV	Backyard chicken	2017	V	Alberta	MT639615	No
AB-S77-ILTV	Backyard chicken	2017	V	Alberta	MT639616	No
AB-S80-ILTV	Backyard chicken	2017	V	Alberta	MT639617	No
AB-S84-ILTV	Backyard chicken	2017	V	Alberta	MT639618	No
AB-S85-ILTV	Backyard chicken	2018	V	Alberta	MT639612	No
AB-S87-ILTV	Backyard chicken	2018	V	Alberta	MT639614	No

**Table 2:** Single nucleotide polymorphisms and amino acid change in reference strains and processed samples compared to LT-Blen (CEO genotype) as reference. Changes are recorded as shared with all samples that are classified into a specific genotype or as being present in specific strains.

SNP Position	Reference Nucleotide	Nucleotide Change	Type of Nucleotide Change	AA Change	Reference AA	AA Change	Genotype and/or Isolates with Same Change
34	A	C	Tn **	12	Lys	Gln	All except IV
49	G	A	Tr ***	17	Ala	Thr	K317
50	**C** *	**T**	Tr	17	Ala	Val	VI
126	T	A	Tn	42	Cys	*	VI-99 and CSW-1
139	C	T	Tr	47	Pro	Ser	757/11
265	G	A	Tr	89	Val	Met	VI-99 and CSW-1
314	T	C	Tr	105	Leu	Ser	LJS09
742	A	G	Tr	248	Thr	Ala	VII, VIII, IX except AB-S20-ILTV and S2.816
848	**T**	**C**	Tr	283	Val	Ala	I, II, III, and AB-S11-ILTV and AB-S15-ILTV
1091	T	C	Tr	364	Val	Ala	VI, VII, VIII, IX
1196	**A**	**C**	Tn	399	Gln	Pro	V
1231	**G**	**T**	Tn	411	Gly	Cys	VI, VII, VIII, IX
1399	G	A	Tr	467	Glu	Lys	VI-99 and CSW-1
1487	A	G	Tr	496	Lys	Arg	VII, VIII, IX
1533	**AG**	**GA**	Tr	512	Ala	Thr	VII, VIII, IX
1588	T	C	Tr	530	Trp	Arg	AB-S7-ILTV and 757/11

* Nucleotide changes in bold—same as already published findings [34]. ** Tn—transversion. *** Tr—transition.

## Supplementary Materials

The following are available online at https://www.mdpi.com/2076-2615/10/9/1634/s1, Figure S1: Visualization of PCR product run in a 1% agarose gel of PCR targeting ORF a and b. The amplicon size is 1751 bp (USDA reference genome coordinates 21,703–23,895). The DNA ladder used was 1 kilo base pairs plus (kb+). Lane 1 is the DNA ladder. Lane labeled 2 is the negative control. Lane labeled 3 is a known positive sample to ILTV., Table S1: Background information of samples from AB provided by Agri Food Laboratories, Alberta Agriculture and Forestry. Samples are arranged by year of submission and the names are color-coded as the phylogenetic tree. Table S2: Background information of samples from BC provided by Animal Health Center. Samples are arranged by year of submission and the names are color-coded as the phylogenetic tree. Table S3: Samples propagated in cell culture and embryonated eggs and number of passages. Table S4: Reference strains used in phylogenetic study arranged by country of origin.
