# Supplementary material for: Genotyping of Infectious Laryngotracheitis Virus (ILTV) Isolates from Western Canadian Provinces of Alberta and British Columbia Based on Partial Open Reading Frame (ORF) a and b
Source: Animals (Basel). 2020 Sep 11;10(9):1634. doi: 10.3390/ani10091634 (PMC7552257; doi:10.3390/ani10091634)
Supplement: Supplementary file 1 [file animals-10-01634-s001.pdf]

Table S1. Background information of samples from AB provided by Agri Food Laboratories, Alberta Agriculture and Forestry. Samples are arranged by year of submission and the names are color-coded as the phylogenetic tree.

Sample	Age	Sample Submission Year and Month	Number of birds in the Flock	Number of Birds Affected	Mortality	Clinical Signs	Gross Lesions
AB-S3-ILTV	6–24 months	oct-13	100	90	15	Sneezing; frothy eyes; runny nose; rattle breathing; swollen heads; crusting eyes.	Mild catarrhal tracheitis
AB-S7-ILTV	Unknown	feb-14	28	6	6	Lethargic, puffy eyes, gurgling breathing and found dead	Fibrinonecrotizing laryngotracheitis, catarrhal sinusitis, conjunctivitis
AB-S11-ILTV	12–16 months	feb-14	50	9	9	Sudden deaths	Fibrinonecrotizing laryngotracheitis
AB-S13-ILTV	7 months	jun-14	60	2	2	Sneezing, watery eyes	Catarrhal sinusitis and tracheitis
AB-S15-ILTV	1.5 months	jun-14	250	10	10	Lethargy, gasping, gurgling, and neck stretching	Hemorrhagic tracheitis
AB-S20-ILTV	10 months	mar-15	150	4	4	Puffed-up eyes, difficult breathing, sudden death	Catarrhal laryngotracheitis
AB-S23-ILTV	7–18 months	abr-15	40	9	9	Sneezing, watery eyes	Tracheitis
AB-S31-ILTV	8 months	abr-15	10	10	1	Sneezing, cough, raspy breathing	Catarrhal and hemorrhagic tracheitis
AB-S35-ILTV	5 months	sep-15	120	Unknown	1	Coughing, bubbly eyes, snotty beak	Catarrhal and hemorrhagic tracheitis
AB-S38-ILTV	4 months	sep-15	20	Unknown	1	Lethargy	Mild catarrhal tracheitis

AB-S41-ILTV	18 months	abr-16	56	22	4	Ruffled feathers, crusty eyes	Hemorrhagic and necrotizing laryngotracheitis
AB-S42-ILTV	15 months	may-16	56	22	4	Gasping, gurgling and difficult breathing	Fibrino-hemorrhagic and necrotizing laryngotracheitis
AB-S44-ILTV	12 months	sep-16	20	Unknown	1	Wheezing respiratory sounds	Catarrhal sinusitis and tracheitis
AB-S45-ILTV	6 months	sep-16	50	4	4	Puffed-up eyes with ocular secretions, conjunctivitis	Fibrinonecrotizing laryngotracheitis
AB-S50-ILTV	2.5 months	oct-16	475	400	40	Difficult breathing, neck stretching and gurgling sounds	Fibrinohemorrhagic and necrotizing laryngotracheitis
AB-S53-ILTV	Unknown	dic-16	30	20	2	Breathing difficulties, sneezing	Catarrhal and hemorrhagic tracheitis; catarrhal sinusitis
AB-S54-ILTV	Unknown	dic-16	30	Unknown	2	Lethargy	Necrotizing laryngotracheitis
AB-S55-ILTV	6 months	dic-16	12	Unknown	1	Swollen eyes with discharge and sneezing	catarrhal sinusitis and necrotizing laryngotracheitis
AB-S56-ILTV	Unknown	ene-17	Unknown	Unknown	8	Sneezing, respiratory distress, conjunctivitis	Laryngotracheitis, caseous conjunctivitis and sinusitis
AB-S62-ILTV	24–36 months	mar-17	100	100	100	Breathing difficulties before death	Fibrinonecrotizing tracheitis
AB-S61-ILTV	24 months	mar-17	50	15	15	Gurgling sounds when breathing	Fibrinonecrotizing laryngotracheitis
AB-S63-ILTV	1.5 months	abr-17	150	5	5	Nasal and ocular secretions, facial swelling and depression	Fibrinohemorrhagic laryngotracheitis

AB-S72-ILTV	3 months	jun-17	70	5	5	Breathing difficulties	Mucohemorrhagic laryngotracehitis
AB-S77-ILTV	20 months	sep-17	150	7	4	Puffy swollen eyes, gasping and neck stretching	Catarrhal and necrohemorrhagic laryngotracheitis
AB-S80-ILTV	7 months	dic-17	100	21	21	Gasping, runny eyes and sneezing	Fibrinonecrotizing laryngotracheitis
AB-S84-ILTV	5.5 months	dic-17	50	5	0	Swollen eyes and sneezing	Fibrinonecrotizing laryngotracheitis
AB-T85-ILTV	10 months	ene-18	120	80	80	Lethargy, swollen eyes and gasping	Fibrinonecrotizing laryngotracheitis
AB-S87-ILTV	24 months	feb-18	8	2	2	Breathing difficulties	Catarrhal sinusitis and tracheitis

Table S2. Background information of samples from BC provided by Animal Health Center. Samples are arranged by year of submission and the names are color-coded as the phylogenetic tree.

Sample	Age	Sample Submission Year	Type of Flock	Type of Operation	Flock Size	Clinical Signs and History
CAN/BC-9-3204	38 days	2009	Commercial	Broiler chicken	7000	Submitted fresh broiler tissue for ILT by PCR.
CAN/BC-9-2276	12 weeks	2009	Commercial	Broiler chicken	12,000	Mortality of 50 birds. Noticed gasping and mucus discharge on birds.
CAN/BC-10-1122	11 weeks	2010	Commercial	Layer chicken	45,000	Increased in mortality. Condition suspected: Respiratory.
CAN/BC-12-1949	36 days	2012	Commercial	Broiler chicken	13,000	Submitted fresh broiler trachea and lungs for ILT by virology/PCR and histopathology. Postmortem findings: fibrinohemorrhagic exudate in trachea and red congested lungs.
CAN/BC-14-6034	Between 6 and 10 months	2014	Backyard	Layer chicken	Not available	Submitted two laying hens for postmortem. All seemed to be healthy. Started losing two birds per week. Sound phlegmy (mucus sounds from throat). Eating and drinking normally. Lost 29 birds in the last 7-10 days

Table S3. Samples propagated in cell culture and embryonated eggs and number of passages.

Sample	Method of Propagation	Number of Propagations
AB-S20-ILTV	CELIC [1]	2
AB-S23-ILTV	CAM [2]	2
AB-S45-ILTV	CELIC	1
AB-S61-ILTV	CAM	1
AB-S63-ILTV	CELIC	2
AB-S84-ILTV	CAM	1
AB-S87-ILTV	CAM	1

[1] CELIC: chicken embryo liver cells. [2] CAM: chorioallantoic membrane.

Table S4. Reference strains used in phylogenetic study arranged by country of origin.

Isolates	Origin	Year	ORF a and b Gene Genotyping	Country	GenBank Accession Number
3.26.90	Backyard flock	1990	IV	USA [1]	MF417809
6.48.88	Backyard flock	1988	VII, VIII, IX	USA	MF417810
14.939	Broiler	2014	V	USA	MF417811
1874C5	Broiler	2004	VI	USA	JN542533
J2	Game chickens	2008	VI	USA	MF417808
63140	Broiler	2006	V	USA	JN542536
USDA	Challenge strain	1960	I, II, III	USA	JN542534
81658	Broiler breeder	2010	I, II, III	USA	JN542535
S2.816	Pea fowl	2002	VII, VIII, IX	USA	MF417807
CEO_HPc	CEO [2] high passage	1990	IV	USA	JN580316
CEO_LPc	CEO low passage	1990	IV	USA	JN580317
CEO_TRVX	Vaccine	1983	IV	USA	JN580313
Nobilis Laringovac(R)	CEO vaccine	1975	IV	USA	KP677881
Laryngo-Vac	CEO vaccine	1975	IV	USA	JQ083494
LT-Blen	CEO vaccine	1975	IV	USA	JQ083493
TCO_HP	TCO [3] high passage	1991	I, II, III	USA	JN580314
TCO_LP	TCO low passage	1991	I, II, III	USA	JN580315
TCO-IVAX	Vaccine IVAX	1983	I, II, III	USA	JN580312
VFAR-043	Field Isolate	2014	VI	Peru	MG775218
A20	Vaccine	1966	VII, VIII, IX	Australia	JN596963
ACC78	Broilers/layers	2008	IV	Australia	JN804826
CSW-1	Layer	1970	VI	Australia	JX646899
CL9	Broilers	2008	IV	Australia	JN804827
SA2	Vaccine	1983	VII, VIII, IX	Australia	JN596962
SERVA	European CEO vaccine	2011	IV	Australia	HQ630064
V1-99	Layer	1999	VI	Australia	JX646898
LJS09	Layer	2009	IV	China	JX458822
K317	Layer		IV	China	JX458824
WG	Vaccine	1950	IV	China	JX458823
30678/14/Ko	Commercial flock		IV	Korea	MH937565
0206/14/Ko	Commercial flock		IV	Korea	MH937564
Rus/Ck/Penza/2013/2701		2013	V	Russia	MF405080
757/11	Field Isolate	2011	V	Italy	KP677884
4787/80	Field Isolate	1980	V	Italy	KP677885

[1] USA: United States of America. [2] CEO: Chicken embryo origin. [3] TCO: Tissue culture origin.

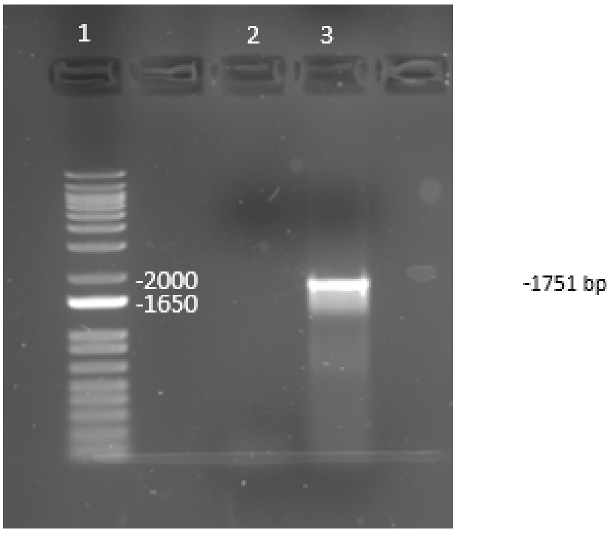

Figure S1. Visualization of PCR product run in a 1% agarose gel of PCR targeting ORF a and b. The amplicon size is 1751 bp (USDA reference genome coordinates 21,703–23,895). The DNA ladder used was 1 kilo base pairs plus (kb+). Lane 1 is the DNA ladder. Lane labeled 2 is the negative control. Lane labeled 3 is a known positive sample to ILTV.
